# Identifying multimorbidity clusters with the highest primary care use: 15 years of evidence from a multi-ethnic metropolitan population

**DOI:** 10.3399/BJGP.2021.0325

**Published:** 2021-11-16

**Authors:** Marina Soley-Bori, Alessandra Bisquera, Mark Ashworth, Yanzhong Wang, Stevo Durbaba, Hiten Dodhia, Julia Fox-Rushby

**Affiliations:** School of Population Health Sciences & Environmental Sciences, King’s College London, London.; School of Population Health & Environmental Sciences, King’s College London, London; National Institute for Health Research (NIHR) Biomedical Research Centre, Guy’s and St Thomas’ NHS Foundation Trust and King’s College London, London.; School of Population Health Sciences & Environmental Sciences, King’s College London, London.; School of Population Health Sciences & Environmental Sciences, King’s College London, London.; School of Population Health & Environmental Sciences, King’s College London, London; NIHR Biomedical Research Centre, Guy’s and St Thomas’ NHS Foundation Trust and King’s College London, London.; Visiting lecturer at King’s College London, London.; School of Population Health & Environmental Sciences, King’s College London, London; NIHR Biomedical Research Centre, Guy’s and St Thomas’ NHS Foundation Trust and King’s College London, London.

**Keywords:** clusters, ethnic group, longitudinal analysis, long-term conditions, multimorbidity, primary care

## Abstract

**Background:**

People with multimorbidity have complex healthcare needs. Some co-occurring diseases interact with each other to a larger extent than others and may have a different impact on primary care use.

**Aim:**

To assess the association between multimorbidity clusters and primary care consultations over time.

**Design and setting:**

A retrospective longitudinal (panel) study design was used. Data comprised electronic primary care health records of 826 166 patients registered at GP practices in an ethnically diverse, urban setting in London between 2005 and 2020.

**Method:**

Primary care consultation rates were modelled using generalised estimating equations. Key controls included the total number of long-term conditions, five multimorbidity clusters, and their interaction effects, ethnic group, and polypharmacy (proxy for disease severity). Models were also calibrated by consultation type and ethnic group.

**Results:**

Individuals with multimorbidity used two to three times more primary care services than those without multimorbidity (incidence rate ratio 2.30, 95% confidence interval = 2.29 to 2.32). Patients in the alcohol dependence, substance dependence, and HIV cluster (Dependence+) had the highest rate of increase in primary care consultations as additional long-term conditions accumulated, followed by the mental health cluster (anxiety and depression). Differences by ethnic group were observed, with the largest impact in the chronic liver disease and viral hepatitis cluster for individuals of Black or Asian ethnicity.

**Conclusion:**

This study identified multimorbidity clusters with the highest primary care demand over time as additional long-term conditions developed, differentiating by consultation type and ethnicity. Targeting clinical practice to prevent multimorbidity progression for these groups may lessen future pressures on primary care demand by improving health outcomes.

## INTRODUCTION

Preventing and managing multimorbidity — the co-occurrence of ≥2 conditions — is challenging for patients, healthcare providers, and policymakers.^1^^–^4 Multimorbidity increases with, but is not confined to, old age,^5^^,^^6^ with a prevalence of 33% among middle- to older-aged adults (37–73 years) in the UK.^7^ People with multimorbidity often experience worse health outcomes^8^^–^12 and use more health services than people without multimorbidity;^13^^–^15 over half of all consultations with GPs in the UK involve patients with multimorbidity.^16^ The clinical heterogeneity of patients with multimorbidity is broad^1^ and some co-occurring diseases interact, changing healthcare costs compared with treating each condition separately.^8^^,^^17^ Calls urge for a transitioning of the health system from specialism in single diseases to patient-focused, cluster-medicine delivery that integrates specialist and generalist care. Research directed at understanding the clustering and sequencing of diseases, and the health and economic impact of multimorbidity, with a view to determining key drivers and efficient interventions is needed.^2^^,^^4^

Most analyses of multimorbidity and primary care use have not been guided by conceptual frameworks^14^ and may disregard relevant confounding variables. Although studies have categorised patients with multimorbidity by disease cluster, none to the authors’ knowledge have included clinical complexity, such as the number and severity of diseases, within the cluster analysis or the impact of different services offered in GP practices.^18^ Disease severity is important as individuals in the same disease cluster may have unique care needs depending on how active their conditions are.^1^^,^^19^^–^21 Part of the complexity behind multimorbidity lies in understanding social factors that enable patients to use health services such as income or ethnic group. Differences in multimorbidity composition and prognosis across ethnic groups have been documented,^22^^–^24 but whether the impact of multimorbidity on primary care needs is magnified for certain ethnic groups remains unknown.^14^ Morbidities tend to accumulate within individuals over time^25^ but relatively few analyses use longitudinal data.^14^^,^^26^ A richer conceptual framework, along with a longitudinal study design, would allow more accurate predictions of utilisation. It should enable healthcare systems to better optimise targeting of preventive care and disease management services.

**Table:** How this fits in

Clinical care for patients with multimorbidity is complex. Understanding which combinations of long-term conditions result in the highest primary care use may inform the targeting of disease prevention and care integration efforts. This study identified the clustering of alcohol dependence, substance dependence, HIV, and mental health conditions as groups associated with the highest increases in primary care demand as additional long-term conditions developed over time. The first estimates, to the authors’ knowledge, of the impact of multimorbidity on primary care consultations across ethnic groups are also provided.

The aim of this study was to assess the association between multimorbidity clusters and primary care consultations over time. Based on a 15-year primary care dataset, a novel conceptual framework and disease clusters identified by Bisquera and colleagues using the same sample,^27^ it pinpoints clusters with the highest use of primary care services, by type and adjusted for a proxy measure of disease severity.

## METHOD

### Study design, setting, and data

A retrospective longitudinal (panel) study design, based on anonymised electronic primary care health records from the Lambeth Data Net, was used. Lambeth, an inner-city borough in south London, contains an urban, deprived, and multiethnic population. The study sample included 826 166 people aged ≥18 years (covering 5 243 478 person–years) who were registered to one of 41 general practices in Lambeth between 1 April 2005 and 31 March 2020. These months align with the quality and outcomes framework (QOF) reporting schedule,^28^ intended to standardise the delivery of primary care.

### Variable selection and specification

Annual primary care consultations per patient per year (1 April to 31 March) were categorised into 14 service types; total consultations, administrative consultations, and 12 combinations of four modes of delivery (face to face, telephone, home visits, and electronic — including email, telemedicine, and text messages), and three provider types (GP, nurse, and other healthcare professional or unspecified clinical — mainly healthcare assistants, physiotherapists, occupational therapist, and pharmacists) (the classification of consultation types into these categories is available from the authors on request). Multimorbidity is defined as having ≥2 of 32 long-term conditions (LTCs) (see Supplementary Appendix S1).^29^

The choice of independent variables was guided by Andersen’s widely used (revised) conceptual framework of healthcare utilisation^30^ and the interrelatedness of comorbidity framework^31^^,^^32^ (see Supplementary Figure S1). Zulman and colleagues postulated that clinical complexity in multimorbidity is directly influenced by the total number of conditions, how comorbidities relate to each other (comorbidity interrelatedness), and their characteristics (for example, symptom intensity and disease severity). The impact of the total number of conditions on clinical complexity may be moderated by their interrelatedness. These four components are represented in this study by the total number of LTCs, five LTC clusters reported by Bisquera *et al* 27 as a measure of LTC interrelatedness, polypharmacy (defined as being prescribed ≥8 medications in different British National Formulary subgroups within a year) as a proxy of disease severity, and an interaction term between the number of LTCs and the clusters.

The five LTC clusters are:

- anxiety and depression (Mental health+);
- heart failure, Parkinson’s disease, osteoporosis, atrial fibrillation, coronary heart disease, chronic kidney disease, stroke or transient ischaemic attack, and dementia (Cardiovascular+);
- osteoarthritis, cancer, chronic pain, hypertension, and diabetes (Pain+);
- chronic liver disease and viral hepatitis (Liver+); and
- alcohol dependence, substance dependence, and HIV (Dependence+).

These groups were identified using multiple correspondence analysis, a statistical technique to analyse clustering of multimorbidity,^33^^,^^34^ and they capture conditions that are as correlated as possible among themselves but not with other groups in the data. The connection reported in previous studies between cardiometabolic diseases and chronic pain; and cardiovascular diseases and dementia for older populations are supported in these clusters too.^25^^,^^27^^,^^35^ Individuals with multimorbidity were assigned to a cluster if >50% of their LTCs belonged to that particular group.

A range of ‘predisposing factors’^30^ that also influence primary care consultations were considered, including categories of self-ascribed ethnic group (specified using the Office for National Statistics 5+1 categories: White, Black (Black/African/Caribbean/Black British), Asian (Asian/Asian British), mixed ethnicity, other, or unknown, age (specific to each year), and sex. ‘Enabling factors’^29^ included poverty, measured through the Index of Multiple Deprivation (IMD-2019 at lower super output area level, stratified into local [Lambeth] and national-based quintiles^36^), and whether their main language spoken was English or not. Age, sex, and ethnic group are self-reported by patients on registration with a GP practice.

### Statistical methods

LTCs, multimorbidity, multimorbidity clusters, polypharmacy, and demographic characteristics are summarised across the study period using means and standard deviations (SDs) for continuous variables, and counts and percentages for categorical variables. Distributions of the 14 primary care consultation rates (total, and by provider type and mode of delivery) are compared between individuals with and without multimorbidity, across multimorbidity clusters, and ethnic groups, based on Mann–Whitney U test for non-normally distributed variables. Missing data are kept as missing.

The relationship between multimorbidity and primary care consultations is assessed using a series of generalised estimating equations with negative binomial distribution and log-link. To account for correlation in repeated measures of the same individual over time, both autoregressive and exchangeable correlation structures are compared using the quasilikelihood under the independence model criterion. Dependent variables selected include total consultations and six broad modes of delivery and provider types (GP, nurse, other healthcare professional, face-to-face, telephone, and home consultations), with patient–year as the unit of analysis. Electronic consultations are limited to descriptive statistics and not modelled separately as they were not available for the whole study period and numbers are insufficient. Administrative consultations are also excluded from modelling as they may reflect contacts unrelated to healthcare need.

The main model specification predicts primary care consultations based on the number of LTCs, indicator variables for each multimorbidity cluster, and a polypharmacy indicator. Interaction effects between the count of LTCs and multimorbidity clusters are tested to assess whether the impact of developing one more of the 32 LTCs varies (or is moderated) by multimorbidity clusters, as suggested by the authors’ conceptual framework. Models also adjust for ethnic group, age, sex, IMD, and language. Year fixed effects are included in the model as covariates. Owing to the interaction term, simple slopes (marginal effect of the number of LTCs across clusters) are computed, and incidence rates generated by exponentiating simple slopes. To illustrate the interaction results, least square means of primary care consultations by number of LTCs and clusters are generated.

As secondary analyses, two additional model specifications are calibrated to facilitate comparisons with previous literature: the first just includes a binary multimorbidity indicator, along with sociodemographic variables; the second adds a polypharmacy indicator to assess the impact of omitting a proxy measure of disease severity on the multimorbidity parameter estimate. For total primary care consultations, the three model specifications are calibrated separately for each ethnic group to assess the variability of multimorbidity effects on primary care consultations by ethnic group.

SAS (version 9.4) was used for all analyses. This study is reported using STROBE guidelines.

## RESULTS

### Population

On average, individuals were registered to a Lambeth practice for mean 5.3 years (SD 4.9), 13% (*n* = 106 896) for less than a year, and 11% (*n* = 91 353) for the entire study period. In total, 60% of the study sample were aged <40 years, 12% were aged ≥60 years (mean 40.39, SD 15.62), and 52% were female. Regarding ethnic group, 54% stated they were of White ethnicity, 14% Black/African/Caribbean/Black British, 6% Asian or Asian British, and 7% mixed ethnicity; with 18% not stating ethnicity. In total, 51% considered English as their main language. Most (65%) lived in socially deprived areas (bottom two quintiles of the national IMD index) (data not shown).

In total, 41% had at least one LTC, and the prevalence of multimorbidity was 21% over time, with an increasing trend from 16% to 25% across the study period. Of the patients with multimorbidity, 38% were in the Mentalhealth+ cluster, 7% the Cardiovascular+ cluster, 33% the Pain+ cluster, 1% the Liver+ cluster, 2% the Dependence+ cluster, and 19% had combinations of conditions that were not highly correlated with any one particular cluster (data not shown).

### Consultations

**Figure 1.**
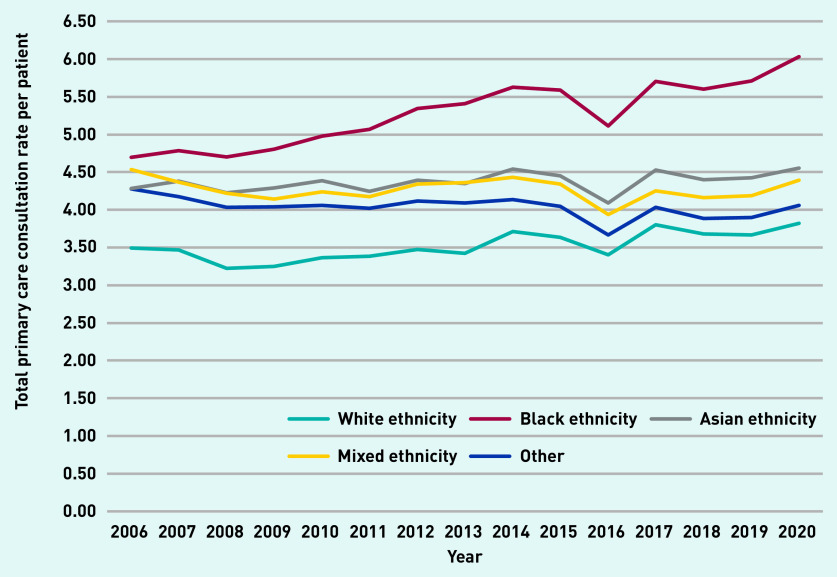
*Total primary care consultation rate by ethnic group: 2006–2020. The 2016 drop is likely because of practice closures with data loss arising as a result of transfer. Categories of self-ascribed ethnic group include White, Black (Black/African/Caribbean/Black British), Asian (Asian/Asian British), mixed ethnicity, other, or unknown.*

Total primary care consultations per year (excluding administrative consultations) and the number of registered patients increased by 61% (1 030 433 to 1 654 076) and 26% (307 157 to 386 238), respectively, from 2006 to 2020 (Table 1). Average consultation rates (per person–year) increased over the study period, from 3.4 (SD 5.4) to 4.3 (SD 6.7) consultations per patient (see Supplementary Figure S2). Individuals of Black ethnicity display a higher and increasing primary care demand over time (Figure 1), with a consultation rate in 2020 of 6.03 (SD 7.8) compared with 3.82 (SD 6.4) for individuals of White ethnicity.

**Table 1.** Total primary care consultations, percentage used by individuals with multimorbidity, and consultation rates for the years 2006, 2013, and 2020^a^

	**2006 (*n*= 307 157)**			**2013 (*n* = 358 427)**			**2020 (*n*= 386 238)**		
	**Total, *n***	**Total used by individuals with multimorbidity, %**	**Consultation rates (per person–year), mean (SD)**	**Total, *n***	**Total used by individuals with multimorbidity, %**	**Consultation rates (per person–year), mean (SD)**	**Total, *n***	**Total used by individuals with multimorbidity, %**	**Consultation rates (per person–year), mean (SD)**
Total consultations	1 030 433	46	3.4 (5.4)	1 438 958	51	4.0 (6.3)	1 654 076	56	4.3 (6.7)
GP face to face	530 345	45	1.7 (3.1)	867 835	51	2.4 (3.8)	811 053	55	2.1 (3.4)
GP telephone	42 030	57	0.1 (0.8)	165 742	56	0.5 (1.6)	349 127	59	0.9 (2.3)
GP home	9821	79	0.03 (0.4)	12 766	89	0.04 (0.5)	14 283	95	0.04 (0.6)
GP electronic	0	—	0	96	63	0.00 (0.0)	2079	48	0.00 (0.1)
Nurse face to face	165 059	44	0.5 (1.6)	248 192	47	0.7 (2.0)	214 515	52	0.6 (1.7)
Nurse telephone	6011	45	0.02 (0.2)	7937	56	0.02 (0.2)	14 437	54	0.04 (0.3)
Nurse home	3507	88	0.01 (0.4)	2245	92	0.01 (0.2)	1916	95	0.01 (0.1)
Nurse electronic	0	—	0	1	100	0.00 (0.0)	87	45	0.0 (0.0)
Other face to face	249 876	43	0.8 (2.2)	128 150	52	0.4 (1.3)	175 469	61	0.5 (1.4)
Other telephone	19 970	49	0.1 (0.5)	5274	55	0.02 (0.2)	69 370	56	0.2 (0.9)
Other home	3814	79	0.01 (0.3)	717	68	0.00 (0.1)	1353	95	0.00 (0.1)
Other electronic	0	—	0	3	67	0.0 (0)	387	46	0.00 (0.0)
Administrative	116 221	45	0.4 (1.4)	164 282	47	0.5 (1.3)	286 255	50	0.7 (1.6)

a *This table excludes intervening years for simplicity. 2006 includes data from April 2005 to March 2006; 2013 includes data from April 2012 to March 2013; and 2020 includes data from April 2019 to March 2020. Descriptives for all data years (2006–2020) are available from the authors on request.* n *indicates that number of registered patients in a given year. Total consultations do not include administrative consultations.*

Individuals with multimorbidity in the unclustered LTCs group showed the highest unadjusted consultation rates in 2020, with an average of 12 primary care consultations (SD 11.8), followed by the Cardiovascular+ cluster (10.7, SD 10.4) and Pain+ cluster (10.6, SD 9.4) (Table 2). Individuals in the unclustered LTCs group had the highest prevalence of obesity (18.9%), chronic obstructive pulmonary disease (16.9%), and epilepsy (10.5%) in this sample (data not shown). The highest rates of home visits were observed in the Cardiovascular+ cluster. Differences in the number of LTCs, per cent of individuals with polypharmacy, age, and ethnic group are observed across clusters (Table 2).

**Table 2.** Primary care consultation rates and sample characteristics by multimorbidity cluster for year 2020^a^

	**Mental health+**	**Cardiovascular+**	**Pain+**	**Liver+**	**Dependence+**	**Unclustered LTCs**	**No multimorbidity**
*n*	67 040	12 017	57 747	1369	3299	32 921	651 773
Total consultations, mean (SD)	6.6 (7.4)	10.7 (10.4)	10.6 (9.4)	4.8 (6.4)	5.3 (6.9)	12.0 (11.8)	2.5 (4.1)
GP consultations, mean (SD)	5.1 (6.1)	7.4 (8.2)	7.2 (7.1)	3.2 (4.8)	3.7 (5.3)	8.6 (9.2)	1.8 (3.1)
Nurse consultations, mean (SD)	0.7 (1.6)	1.4 (3.5)	1.6 (3.1)	0.7 (1.7)	0.8 (2.6)	1.6 (3.8)	0.4 (1.1)
Other consultations, mean (SD)	0.8 (1.9)	1.9 (3.4)	1.9 (3.1)	0.8 (2.1)	0.8 (2.0)	1.9 (3.4)	0.3 (1.1)
Face-to-face consultations, mean (SD)	4.7 (5.2)	7.0 (6.9)	7.9 (6.9)	3.6 (4.8)	3.9 (5.4)	8.2 (8.0)	1.9 (3.1)
Telephone consultations, mean (SD)	1.9 (3.4)	3.0 (4.1)	2.5 (4.1)	1.1 (2.4)	1.3 (2.6)	3.5 (5.7)	0.6 (1.6)
Home consultations, mean (SD)	0.0 (0.3)	0.7 (2.4)	0.2 (1.3)	0.0 (0.2)	0.0 (0.3)	0.3 (1.5)	0.0 (0.1)
Number of LTCs, mean (SD)	2.5 (0.7)	3.9 (2.1)	3.5 (1.6)	2.3 (0.6)	2.6 (0.9)	4.6 (2.1)	0.2 (0.4)
With polypharmacy, %	4.0	24.4	18.6	3.6	3.8	20.6	0.6
Age, years, mean (SD)	39.7 (12.6)	76.1 (15.5)	64.0 (16.5)	44.6 (11.5)	41.7 (11.2)	55.6 (18.9)	36.9 (12.2)
White ethnicity, %	65.4	56.5	43.1	43.5	64.2	60.5	53.3

a *2020 includes data from April 2019 to March 2020. Consultation rate descriptives are similar across time and are available from the authors on request for all data years. Mental health+ includes anxiety and depression; Cardiovascular+ includes heart failure, Peripheral Arterial Disease (PAD), osteoporosis, atrial fibrillation, coronary heart disease, chronic kidney disease, stroke/transient ischaemic attack, and dementia; Pain+ includes osteoarthritis, cancer, chronic pain, hypertension, and diabetes; Liver+ includes chronic liver disease and viral hepatitis; Dependence+ includes alcohol dependence, substance dependence, and HIV; Unclustered LTCs include: Parkinson’s disease, chronic obstructive pulmonary disease, asthma, inflammatory bowel disease, lupus, multiple sclerosis, rheumatoid arthritis, morbid obesity, cognitive and learning disabilities, sickle-cell anaemia, serious mental illness, and epilepsy. LTC = long-term condition. SD = standard deviation.*

Among individuals with multimorbidity, those of Black ethnicity (10.3, SD 9.5), Asian ethnicity (10.4, SD 10.0), or multiple ethnicities (9.2, SD 9.3) showed higher total primary care consultations rates than individuals of White ethnicity (8.7, SD 9.5) (data not shown).

### Main results

The impact of developing one more LTC on primary care consultations varies by multimorbidity clusters (*P*<0.0001 for the number of LTCs × cluster interaction) (Table 3, model 1). The highest rate of increase for every type of primary care visit (except for nurses) occurs in the Dependence+ cluster, followed by the MentalHealth+ cluster. For nurse primary care consultations, the Liver+ cluster shows the largest rate of increase when an additional LTC arises. Supplementary Figure S3 illustrates the differences in estimated marginal mean rates.

**Table 3.** Adjusted IRR of multimorbidity-related variables by primary care consultation type across three model specifications^a^

	**IRR (95% CI)**						

	**Total**	**GP**	**Nurse**	**Other**	**Face to face**	**Telephone**	**Home**
**Model 1: multimorbidity clusters, count LTCs, interaction, and polypharmacy**							
Mental health+	1.24 (1.23 to 1.24)	1.24 (1.23 to 1.25)	1.12 (1.11 to 1.14)	1.26 (1.24 to 1.28)	1.22 (1.21 to 1.23)	1.28 (1.26 to 1.30)	1.33 (1.29 to 1.38)
Cardiovascular+	1.10 (1.09 to 1.10)	1.10 (1.10 to 1.11)	1.05 (1.04 to 1.07)	1.10 (1.08 to 1.11)	1.06 (1.06 to 1.07)	1.18 (1.17 to 1.19)	1.23 (1.21 to 1.24)
Pain+	1.11 (1.10 to 1.11)	1.11 (1.11 to 1.11)	1.08 (1.07 to 1.09)	1.12 (1.11 to 1.13)	1.09 (1.09 to 1.09)	1.17 (1.16 to 1.18)	1.24 (1.23 to 1.25)
Liver+	1.22 (1.14 to 1.29)	1.20 (1.12 to 1.28)	1.27 (1.13 to 1.43)	1.23 (1.06 to 1.43)	1.20 (1.13 to 1.27)	1.37 (1.21 to 1.55)	1.20 (0.96 to 1.49)
Dependence+	1.33 (1.29 to 1.37)	1.35 (1.32 to 1.40)	1.14 (1.03 to 1.26)	1.41 (1.33 to 1.49)	1.30 (1.26 to 1.35)	1.48 (1.40 to 1.55)	1.36 (1.25 to 1.48)
Unclustered LTCs	1.11 (1.10 to 1.11)	1.11 (1.10 to 1.11)	1.07 (1.06 to 1.08)	1.13 (1.12 to 1.14)	1.09 (1.08 to 1.09)	1.16 (1.15 to 1.16)	1.17 (1.16 to 1.18)

**Model 2: multimorbidity only**							
Multimorbidity (yes)	2.64 (2.63 to 2.66)	2.74 (2.72 to 2.75)	2.28 (2.27 to 2.32)	2.68 (2.64 to 2.69)	2.56 (2.53 to 2.59)	3.16 (3.10 to 3.19)	5.47 (5.37 to 5.58)

**Model 3: multimorbidity and polypharmacy**							
Multimorbidity (yes)	2.30 (2.29 to 2.32)	2.36 (2.36 to 2.49)	1.92 (1.90 to 1.93)	2.36 (2.34 to 2.39)	2.24 (2.20 to 2.25)	2.61 (2.59 to 2.64)	3.83 (3.74 to 3.91)
Polypharmacy (yes)	2.20 (2.18 to 2.21)	2.29 (2.27 to 2.30)	2.36 (2.32 to 2.39)	1.95 (1.93 to 1.97)	2.16 (2.14 to 2.16)	2.53 (2.51 to 2.56)	4.04 (3.97 to 4.10)

a n *= 5 243 478 person–years, corresponding to 826 166 individuals. Data from April 2005 to March 2020 are used. All models also adjust for age, sex, ethnic group, Index of Multiple Deprivation quintiles, and language. Multimorbidity clusters: Mental health+ includes anxiety and depression; Cardiovascular+ includes heart failure, Peripheral Arterial Disease (PAD), osteoporosis, atrial fibrillation, coronary heart disease, chronic kidney disease, stroke/transient ischaemic attack, and dementia; Pain+ includes osteoarthritis, cancer, chronic pain, hypertension, and diabetes; Liver+ includes chronic liver disease and viral hepatitis; Dependence+ includes alcohol dependence, substance dependence, and HIV; Unclustered LTCs include: Parkinson’s disease, chronic obstructive pulmonary disease, asthma, inflammatory bowel disease, lupus, multiple sclerosis, rheumatoid arthritis, morbid obesity, cognitive and learning disabilities, sickle-cell anaemia, serious mental illness, and epilepsy. The reference category for both multimorbidity and multimorbidity clusters is not having multimorbidity. Categories of self-ascribed ethnic group include White, Black (Black/African/Caribbean/Black British), Asian (Asian/Asian British), mixed ethnicity, other, or unknown. In model 3, the count of LTCs and multimorbidity clusters are included as main effects, along with an interaction between the two variables. Parameter estimates of the main effects cannot be interpreted by themselves anymore because of the interaction. Simple slopes (marginal effect of the continuous variable — number of LTCs — across the different levels of the categorical variable–clusters) are computed instead, and incidence rates generated by exponentiating simple slopes. For example, in model 3, the IRR for each cluster indicates the effect of developing one more LTC for individuals in that specific cluster. For the Dependence+ cluster, IRR 1.33, so for a one unit increase in the number of LTCs, the incidence rate of primary care consultations increases by 33%, while in the Cardiovascular+ cluster it increases by 10%. IRR = incidence rate ratio. LTC = long-term condition.*

Primary care consultations show a particularly large predicted increase among individuals with complex multimorbidity (≥3 LTCs) in the Dependence+ cluster as additional LTCs accumulate.

Regarding the impact of predisposing and enabling factors on primary care use, primary care consultations increased with age, particularly home visits. The total primary care consultation incidence rate is 18% higher among individuals between 60 and 79 years compared with those between 18 and 39 years of age (incidence rate ratio [IRR] 1.18, 95% confidence interval [CI] = 1.18 to 1.21), and 4.32 times higher for home consultations (IRR 4.32, 95% CI = 4.20 to 4.45). Female patients tended to use primary care services more often than male patients (IRR 1.66, 95% CI = 1.66 to 1.67 for total consultations), and the most deprived individuals consulted slightly more than the least deprived individuals, except for nurse and phone consultations. Individuals from ethnic groups other than White (Black, Asian, mixed, or other) were more likely to use primary care services than individuals of White ethnicity, except for home visits. The largest effect is observed among those of Black ethnicity (IRR 1.17, 95% CI = 1.16 to 1.17 for total consultations, and IRR 0.74, 95% CI = 0.73 to 0.76 for home consultations). Language is also significantly associated with primary care consultations, except for face-to-face visits (see Supplementary Table S1).

Models 2 and 3 (Table 3), with multimorbidity as a binary indicator, indicate that multimorbidity is associated with an increase in all types of primary care consultation rates, with the largest effect observed for home visits (Table 3, model 2: IRR 2.64, 95% CI = 2.63 to 2.66 for total consultations and IRR 5.47, 95% CI = 5.37 to 5.58 for home visits). When polypharmacy is added to the model (Table 3, model 3), the association with multimorbidity decreases but remains large (IRR 2.30, 95% CI = 2.29 to 2.32, and IRR 3.83, 95% CI = 3.74 to 3.91 for home visits). The stratification of the total primary care consultations models by ethnic group reveals some variability in the effect of multimorbidity across ethnic groups (from IRR 2.13, 95% CI = 2.11 to 2.15 for Black ethnicity to 2.49, 95% CI = 2.41 to 2.57 for other ethnicity) (Table 4, model 3). The largest rate of increase in total primary consultations when an additional LTC developed was observed in the Liver+ cluster for individuals of Black or Asian ethnicity, whereas Dependence+ remained the cluster with the largest impact among the other ethnic groups (Table 4, model 1).

**Table 4.** Adjusted IRR of multimorbidity-related variables predicting total primary care consultations, across three model specifications and ethnic group^a^

	**IRR (95% CI)**					

	**All**	**White Ethnicity**	**Black Ethnicity**	**Asian Ethnicity**	**Mixed Ethnicity**	**Other**
Individuals (person-years)	826 166 (5 243 478)	445 460 (2 724 461)	113 722 (960 700)	49 893 (327 250)	31 197 (202 016)	23 727 (144 416)

**Model 1: multimorbidity clusters, count LTCs, interaction, and polypharmacy**						
Mental health+	1.24 (1.23 to 1.24)	1.24 (1.23 to 1.25)	1.19 (1.17 to 1.21)	1.23 (1.19 to 1.27)	1.23 (1.19 to 1.26)	1.24 (1.19 to 1.29)
Cardiovascular+	1.10 (1.09 to 1.10)	1.09 (1.08 to 1.10)	1.12 (1.10 to 1.13)	1.10 (1.08 to 1.12)	1.13 (1.10 to 1.16)	1.09 (1.03 to 1.14)
Pain+	1.11 (1.10 to 1.11)	1.11 (1.10 to 1.11)	1.11 (1.11 to 1.12)	1.10 (1.09 to 1.11)	1.11 (1.09 to 1.13)	1.13 (1.11 to 1.15)
Liver+	1.22 (1.14 to 1.29)	1.15 (1.05 to 1.26)	1.40 (1.28 to 1.52)	1.41 (1.16 to 1.71)	1.06 (0.78 to 1.44)	1.19 (0.97 to 1.45)
Dependence+	1.33 (1.29 to 1.37)	1.33 (1.28 to 1.37)	1.30 (1.30 to 1.21)	1.28 (1.04 to 1.59)	1.33 (1.18 to 1.50)	1.42 (1.20 to 1.68)
Unclustered LTCs	1.11 (1.10 to 1.11)	1.10 (1.09 to 1.10)	1.12 (1.11 to 1.12)	1.11 (1.09 to 1.12)	1.11 (1.10 to 1.13)	1.14 (1.11 to 1.17)

**Model 2: multimorbidity only**						
Multimorbidity (yes)	2.64 (2.63 to 2.66)	2.58 (2.56 to 2.60)	2.46 (2.43 to 2.49)	2.77 (2.71 to 2.83)	2.56 (2.50 to 2.62)	2.95 (2.85 to 3.06)

**Model 3: multimorbidity and polypharmacy**						
Multimorbidity (yes)	2.30 (2.29 to 2.32)	2.22 (2.21 to 2.24)	2.13 (2.11 to 2.15)	2.31 (2.27 to 2.36)	2.22 (2.17 to 2.27)	2.49 (2.41 to 2.57)
Polypharmacy (yes)	2.20 (2.18 to 2.21)	2.29 (2.28 to 2.31)	2.04 (2.02 to 2.06)	2.17 (2.14 to 2.21)	2.25 (2.20 to 2.31)	2.31 (2.23 to 2.39)

a n *= 5 243 478 person–years, corresponding to 826 166 individuals. Data from April 2005 to March 2020 are used. All models also adjust for age, sex, ethnic group, Index of Multiple Deprivation quintiles, and language. Multimorbidity clusters: Mental health+ includes anxiety and depression; Cardiovascular+ includes heart failure, Peripheral Arterial Disease (PAD), osteoporosis, atrial fibrillation, coronary heart disease, chronic kidney disease, stroke/transient ischaemic attack, and dementia; Pain+ includes osteoarthritis, cancer, chronic pain, hypertension, and diabetes; Liver+ includes chronic liver disease and viral hepatitis; Dependence+ includes alcohol dependence, substance dependence, and HIV; Unclustered LTCs include: Parkinson’s disease, chronic obstructive pulmonary disease, asthma, inflammatory bowel disease, lupus, multiple sclerosis, rheumatoid arthritis, morbid obesity, cognitive and learning disabilities, sickle-cell anaemia, serious mental illness, and epilepsy. The reference category for both multimorbidity and multimorbidity clusters is not having multimorbidity. Categories of self-ascribed ethnic group include White, Black (Black/African/Caribbean/Black British), Asian (Asian/Asian British), mixed ethnicity, other, or unknown. In model 3, the count of LTCs and multimorbidity clusters are included as main effects, along with an interaction between the two variables. Parameter estimates of the main effects cannot be interpreted by themselves anymore because of the interaction. Simple slopes (marginal effect of the continuous variable — number of LTCs — across the different levels of the categorical variable–clusters) are computed instead, and incidence rates generated by exponentiating simple slopes. For example, in model 3, the IRR for each cluster indicates the effect of developing one more LTC for individuals in that specific cluster. For the Dependence+ cluster, IRR 1.33, so for a one unit increase in the number of LTCs, the incidence rate of primary care consultations increases by 33%, while in the Cardiovascular+ cluster it increases by 10%. IRR = incidence rate ratio. LTC = long-term conditions.*

Parameter estimates remained stable when year 2016 was removed from the study sample and when clinic fixed effects were added (results available from the authors on request). A drop in total consultations was observed in 2016 (see Supplementary Figure S2), likely because of practice closures with data loss arising as a result of transfer.

## DISCUSSION

### Summary

This study assessed the longitudinal effects of multimorbidity clusters on primary care consultations among an ethnically diverse and, predominantly, working-age population in south London between 2005 and 2020. The findings indicate that the Dependence+ cluster, followed by the Mentalhealth+ cluster, show the largest rate of increase when an additional LTC develops for all consultation types except for nurse consultations, where the Liver+ cluster had the largest impact. Some variability across ethnic groups is reported, with the largest rate of increase on total primary consultations due to an additional LTC in the Liver+ cluster for individuals of Black or Asian ethnicity, while Dependence+ remained the cluster with the largest impact among the other ethnic groups.

### Comparison with existing literature

Results from the simplest specification — model 1, with presence of multimorbidity — align with previous findings that multimorbidity more than doubles primary care consultations.^16^ The cluster results in the current study add to the sparse literature on multimorbidity clusters and primary care consultations, and both mirror and challenge existing findings. For example, Zhu *et al* found the highest utilisation among individuals in the depression, anxiety, and painful conditions cluster (IRR 3.21, 95% CI = 3.07 to 3.36), followed by the alcohol, psychoactive substance misuse, and painful conditions (IRR 3.12, 95% CI = 2.83 to 3.46) cluster.^18^ These estimates are larger than the current research, which could be explained by differences in study design (cross-sectional), techniques to identify LTC clusters (clustering of individuals rather than LTCs), and model specification (LTC counts and interaction effects were not accounted for in Zhu *et al*). However, both studies point to alcohol and substance dependence, and mental health clusters as important drivers of primary care consultations in patients with multimorbidity.

Stokes *et al* found no clear multimorbidity combinations based on secondary care costs rather than LTC prevalence and co-occurrence.^37^ This alternative approach to clustering, motivated by directly identifying the most expensive combinations to inform cost-saving interventions, warrants further exploration as an alternative to current clustering methodologies for primary care data.

### Strengths and limitations

Model specification was grounded in two conceptual frameworks that guided a refined specification of the clinical complexity of multimorbidity and a careful consideration of confounders in econometric analyses. This study, to the authors’ knowledge, provides the first analyses of the interplay between disease severity and multimorbidity as well as differences across ethnic groups. Disaggregating total primary care consultations by provider type and mode of delivery allowed a more nuanced characterisation of the healthcare demand of individuals with multimorbidity. A unique strength of this study is the use of a 15-year dataset, rich with an ethnically diverse population that improves accuracy of parameter estimates because of larger sample variability and allows the exploration of trends over time compared with cross-sectional data. Results may not be generalisable to rural, less ethnically diverse, or older populations. Other factors that may explain primary care consultations could not be measured, such as social support (for example, marital status) and further multimorbidity characteristics (clinical dominance or time since diagnosis). Disease severity was measured through polypharmacy, which may fail to capture important aspects of disease progression not always linked to medications such as functional impairment. Primary care use may also increase the probability of diagnosing LTC, therefore is it difficult to determine the direction of causality between the number of conditions and consultations. Additional LTCs may also be diagnosed from the regular monitoring of the index LTC. Finally, recording accuracy may vary across the 32 LTCs, with a likely under-recording of non-QOF conditions.

### Implications for research and practice

Understanding the health and social care needs of patients with multimorbidity is required to effectively transition from a single-disease to a cluster-medicine oriented delivery model.^38^ This research provides evidence in support of this policy goal by identifying disease clusters associated with the highest primary care use, differentiating across consultation types, and ethnic groups.

The impact of multimorbidity clusters on total costs including primary, secondary care, and social care remains unknown. Bringing together data across the care continuum is needed to fully characterise the multimorbidity journey and identify LTCs that most commonly lead to the highest multimorbidity clinical complexity, worst health outcomes, and costly care pathways. Little is known about the expected trajectories of health service use by the most prevalent disease clusters as LTCs accumulate, and how these trajectories differ across ethnic groups. Finally, research on the effectiveness and cost-effectiveness of interventions aimed at preventing and improving the management of the multimorbidity journey for individuals at the highest risk of accumulating the most expensive LTCs is needed.^39^

In conclusion, this study identified the clustering of alcohol dependence, substance dependence, HIV, and also the clustering of mental health conditions as groups associated with the highest increases in primary care demand as additional LTCs develop. Designing and implementing payment incentives to target primary care interventions to these individuals to prevent further acquisition of diseases may improve their health outcomes and reduce future primary care use.

## Data

The data are not publicly available to share, but the research group can provide descriptive data in table form. Requests should be made to Mark Ashworth (mark. ashworth@kcl.ac.uk).

## References

[1] Academy of Medical Sciences (2018). Multimorbidity: a priority for global health research.

[2] Pearson-Stuttard J, Ezzati M, Gregg EW (2019). Multimorbidity — a defining challenge for health systems. Lancet Public Health.

[3] Chiolero A, Rodondi N, Santschi V (2020). High-value, data-informed, and team-based care for multimorbidity. Lancet Public Health.

[4] Whitty CJM, MacEwen C, Goddard A (2020). Rising to the challenge of multimorbidity.. BMJ.

[5] Barnett K, Mercer SW, Norbury M (2012). Epidemiology of multimorbidity and implications for health care, research, and medical education: a cross-sectional study. Lancet.

[6] Prados-Torres A, Poblador-Plou B, Gimeno-Miguel A (2018). Cohort profile: the epidemiology of chronic diseases and multimorbidity. The EpiChron cohort study. Int J Epidemiol.

[7] Jani BD, Hanlon P, Nicholl BI (2019). Relationship between multimorbidity, demographic factors and mortality: findings from the UK Biobank cohort. BMC Med.

[8] Angelantonio ED, Kaptoge S, Emerging Risk Factors Collaboration (2015). Association of cardiometabolic multimorbidity with mortality. JAMA.

[9] Li J, Green M, Kearns B (2016). Patterns of multimorbidity and their association with health outcomes within Yorkshire, England: baseline results from the Yorkshire Health Study. BMC Public Health.

[10] Vetrano DL, Rizzuto D, Calderón-Larrañaga A (2018). Trajectories of functional decline in older adults with neuropsychiatric and cardiovascular multimorbidity: a Swedish cohort study. PLOS Med.

[11] Dumbreck S, Flynn A, Nairn M (2015). Drug–disease and drug–drug interactions: systematic examination of recommendations in 12 UK national clinical guidelines.. BMJ.

[12] McCoy RG, Lipska KJ, Houten HKV, Shah ND (2020). Association of cumulative multimorbidity, glycemic control, and medication use with hypoglycemia-related emergency department visits and hospitalizations among adults with diabetes. JAMA Netw Open.

[13] Lehnert T, Heider D, Leicht H (2011). Review: health care utilization and costs of elderly persons with multiple chronic conditions. Med Care Res Rev.

[14] Soley-Bori M, Ashworth M, Bisquera A (2021). Impact of multimorbidity on healthcare costs and utilisation: a systematic review of the UK literature. Br J Gen Pract.

[15] Palladino R, Pennino F, Finbarr M (2019). Multimorbidity and health outcomes in older adults in ten European health systems, 2006–15. Health Aff (Millwood).

[16] Cassell A, Edwards D, Harshfield A (2018). The epidemiology of multimorbidity in primary care: a retrospective cohort study. Br J Gen Pract.

[17] Brilleman SL, Purdy S, Salisbury C (2013). Implications of comorbidity for primary care costs in the UK: A retrospective observational study. Br J Gen Pract.

[18] Zhu Y, Edwards D, Mant J (2020). Characteristics, service use and mortality of clusters of multimorbid patients in England: a population-based study. BMC Med.

[19] Salisbury C, Johnson L, Purdy S (2011). Epidemiology and impact of multimorbidity in primary care: a retrospective cohort study. Br J Gen Pract.

[20] Payne RA, Abel GA, Guthrie B, Mercer SW (2013). The effect of physical multimorbidity, mental health conditions and socioeconomic deprivation on unplanned admissions to hospital: a retrospective cohort study. CMAJ.

[21] Zulman DM, Chee CP, Wagner TH (2015). Multimorbidity and healthcare utilisation among high-cost patients in the US Veterans Affairs Health Care System. BMJ Open.

[22] Quiñones AR, Botoseneanu A, Markwardt S (2019). Racial/ethnic differences in multimorbidity development and chronic disease accumulation for middle-aged adults. PLoS One.

[23] Kalgotra P, Sharda R, Croff JM (2020). Examining multimorbidity differences across racial groups: a network analysis of electronic medical records. Sci Rep.

[24] Quiñones AR, Allore HG, Botoseneanu A (2020). Tracking multimorbidity changes in diverse racial/ethnic populations over time: issues and considerations. J Gerontol Ser A.

[25] Ashworth M, Durbaba S, Whitney D (2019). Journey to multimorbidity: longitudinal analysis exploring cardiovascular risk factors and sociodemographic determinants in an urban setting. BMJ Open.

[26] France EF, Wyke S, Gunn JM (2012). Multimorbidity in primary care: a systematic review of prospective cohort studies. Br J Gen Pract.

[27] Bisquera A, Gulliford M, Dodhia H (2021). Identifying longitudinal clusters of multimorbidity in an urban setting: a population-based cross-sectional study. Lancet Reg Health.

[28] NHS Digital (2020). Quality and Outcomes Framework (QOF) business rules v 38 2017–2018 October code release. https://digital.nhs.uk/data-and-information/data-collections-and-data-sets/data-collections/quality-and-outcomesframework-qof/quality-and-outcome-framework-qof-business-rules/quality-and-outcomes-framework-qof-business-rules-v-38-2017-2018-october-coderelease.

[29] Hafezparast N, Turner EB, Dunbar-Rees R (2021). Adapting the definition of multimorbidity — development of a locality-based consensus for selecting included long term conditions. BMC Fam Pract.

[30] Andersen RM (1995). Revisiting the behavioral model and access to medical care: does it matter?. J Health Soc Behav.

[31] Zulman DM, Asch SM, Martins SB (2014). Quality of care for patients with multiple chronic conditions: the role of comorbidity interrelatedness. J Gen Intern Med.

[32] Bowling CB, Plantinga L, Phillips LS (2017). Association of multimorbidity with mortality and healthcare utilization in chronic kidney disease. J Am Geriatr Soc.

[33] García-Olmos L, Salvador CH, Alberquilla Á (2012). Comorbidity patterns in patients with chronic diseases in general practice. PLoS One.

[34] Guisado-Clavero M, Roso-Llorach A, López-Jimenez T (2018). Multimorbidity patterns in the elderly: a prospective cohort study with cluster analysis. BMC Geriatr.

[35] Mathur R, Hull SA, Badrick E, Robson J (2011). Cardiovascular multimorbidity: the effect of ethnicity on prevalence and risk factor management. Br J Gen Pract.

[36] Ministry of Housing, Communities & Local Government (2019). English indices of deprivation 2019. https://www.gov.uk/government/statistics/englishindices-of-deprivation-2019.

[37] Stokes J, Guthrie B, Mercer SW (2021). Multimorbidity combinations, costs of hospital care and potentially preventable emergency admissions in England: a cohort study. PLoS Med.

[38] NHS England (2019). Next steps on the NHS five year forward view.

[39] Kastner M, Cardoso R, Lai Y (2018). Effectiveness of interventions for managing multiple high-burden chronic diseases in older adults: a systematic review and meta-analysis. CMAJ.

